# Single point insulin sensitivity estimator index and incident impaired fasting glucose in Chinese adults: a retrospective cohort study

**DOI:** 10.3389/fendo.2026.1810066

**Published:** 2026-06-05

**Authors:** Duo Yang, Renzhe Lin, Sen Li, Shujun Ye, Zitian Luo, Huankai Zhang, Si Wu, Longsheng Zhang

**Affiliations:** 1 Department of Anesthesiology, Jieyang People’s Hospital, Jieyang, Guangdong, China; 2 First Clinical Medical College, Guangdong Medical University, Zhanjiang, Guangdong, China

**Keywords:** adults, body mass index, diabetes, endocrinology, impaired fasting glucose, insulin sensitivity, predictive markers, single point insulin sensitivity estimator

## Abstract

**Background:**

Insulin resistance is the core pathophysiological mechanism underlying the development of impaired fasting glucose (IFG) and diabetes mellitus. The SPISE index is a novel, simple surrogate marker of insulin sensitivity that does not require insulin measurement. However, the predictive value of the SPISE index for IFG remains to be further elucidated. This study aimed to investigate the association between the baseline SPISE index and the risk of incident IFG using retrospective longitudinal cohort data from Chinese adults.

**Methods:**

A total of 100494 Chinese adults with normal fasting plasma glucose at baseline were included. The exposure was the SPISE index, and the primary outcome was incident IFG during follow-up. Multivariable Cox proportional hazards regression models and logistic regression models were employed to assess the associations. RCS was used to explore the relationship between the SPISE index and incident IFG. Extensive subgroup and sensitivity analyses were performed to verify the robustness of the results.

**Results:**

During a median follow-up of 2.99 years, the incidence of IFG in the overall study population was 12.33%. A higher SPISE index was independently associated with a lower risk of IFG. In the fully adjusted Cox model, each one-unit increase in the SPISE index was associated with a hazard ratio of 0.91 for incident IFG. Participants in the Q4 had an approximately 40% lower risk of developing IFG compared to those in the Q1, showing a significant dose-response relationship. RCS analysis indicated a linear inverse association between the SPISE index and IFG risk. Subgroup and sensitivity analyses confirmed the consistency and robustness of this association across different population characteristics and data handling approaches.

**Conclusions:**

In a community-based Chinese adult population with normal baseline blood glucose, a lower SPISE index is an independent risk factor for future incident IFG, with a linear inverse dose-response relationship. Our findings suggest that the SPISE index could potentially be implemented in routine clinical practice as a simple and cost-effective tool for identifying individuals at high risk for IFG, serving as an effective tool for the early identification of high-risk individuals for IFG and for implementing targeted primary prevention strategies for diabetes.

## Background

Diabetes Mellitus (DM) has emerged as one of the most critical global public health challenges of the 21st century. Characterized by a rapidly escalating prevalence and widespread impact, it imposes a substantial burden on healthcare systems worldwide. According to the latest data from the International Diabetes Federation (IDF), approximately 537 million adults worldwide are currently living with DM. With the prevalence continuing to rise, the total number of patients is projected to reach 783 million by 2045 and approach 900 million by 2050 (1, 2). China harbors the world’s largest DM population, comprising over 118 million individuals—approximately 22% of the global total (3, 4). Ranking first globally in disease burden, coupled with a vast number of undiagnosed cases, China faces an extremely severe challenge in the prevention and control of DM. The spectrum of microvascular and macrovascular complications resulting from DM—including retinopathy, nephropathy, neuropathy, and cardiovascular/cerebrovascular diseases—not only severely impairs patients’ quality of life but also serves as a major cause of disability and mortality, thereby generating a colossal socioeconomic burden (5). Impaired fasting glucose (IFG), a critical state of prediabetes, is considered an obligatory transitional stage from normal glucose homeostasis to DM. It is defined by fasting plasma glucose (FPG) levels that are elevated within a specific range but remain below the diagnostic threshold for DM (6, 7). Research indicates that individuals with IFG are not only prone to progressing to DM in the coming years but also face a significantly increased risk of cardiovascular disease (8, 9). Consequently, IFG can be regarded as a pivotal “early warning window.” In this context, the early identification of individuals at high risk for IFG is of paramount importance. Effective screening and risk stratification of this population can provide a critical window for intervention. Evidence suggests that intensive lifestyle interventions or pharmacological strategies implemented during the prediabetic stage can significantly delay or prevent the onset of DM (10, 11). Therefore, identifying accurate, convenient, and scalable tools for IFG risk prediction is fundamental to the implementation of primary prevention strategies for DM and holds substantial clinical significance for curbing the epidemic trend of DM in China.

Insulin resistance (IR) and the subsequent decompensation of pancreatic β-cell function constitute the central pathophysiological mechanisms underlying the onset and progression of IFG and DM (12, 13). IR is defined as a diminished biological response to insulin, resulting in reduced efficiency of insulin-mediated glucose uptake and utilization. To maintain glucose homeostasis, pancreatic β-cells compensatorily secrete excessive insulin, leading to hyperinsulinemia. This compensatory mechanism eventually fails, precipitating hyperglycemia that first manifests as IFG and subsequently progresses to DM (14, 15). Consequently, IR serves not only as the initiating factor for IFG but also as a critical nexus linking obesity and metabolic syndrome to cardiovascular disease (16). Currently, the hyperinsulinemic-euglycemic clamp is considered the “gold standard” for assessing IR, as it allows for the direct and quantitative measurement of whole-body insulin sensitivity (17). However, its complexity, time-intensive nature, high cost, and requirement for specialized personnel render it unsuitable for large-scale epidemiological surveys or routine clinical practice (18). Although surrogate indices based on insulin concentrations derived from fasting or oral glucose tolerance tests (OGTT)—such as the Homeostatic Model Assessment for Insulin Resistance (HOMA-IR) and the Matsuda index—have simplified assessment and are widely utilized (19), they depend on accurate insulin quantification. Insulin immunoassays are plagued by a lack of standardization, significant inter-assay variability, and relatively high costs, limiting their utility in primary care settings and large-scale public health initiatives. Therefore, the identification of an effective tool for assessing IR that is independent of insulin measurement—relying instead on routinely available and highly standardized metabolic parameters—holds significant practical value for population-level IR screening and IFG risk prediction (20).

In recent years, researchers have actively sought novel surrogate markers for IR that are independent of insulin measurement. The Single Point Insulin Sensitivity Estimator (SPISE) has emerged as a promising tool in this regard; its calculation relies solely on three routine, standardized, and cost-effective parameters: fasting high-density lipoprotein cholesterol (HDL-C), triglycerides (TG), and body mass index (BMI) (21). The advantage of the SPISE index lies in its integration of key components of the pathophysiological process of IR: low HDL-C and high TG levels are core manifestations of insulin-resistant dyslipidemia, while BMI serves as a classic indicator of general obesity and metabolic burden. This index was originally validated by Paulmichl et al. in 2016 among white adolescents and adults. Their study demonstrated that the area under the receiver operating characteristic curve (AUROC) for the SPISE index was significantly superior to the TG/HDL-C ratio. Furthermore, when compared against the M-value, the AUROC of the SPISE index was comparable to the Matsuda insulin sensitivity index and equivalent to the Quantitative Insulin Sensitivity Check Index (QUICKI) and the HOMA-IR (21). Subsequently, multiple cross-sectional studies have validated the utility of the SPISE index across diverse populations and disease contexts. For instance, the SPISE index has demonstrated excellent discriminatory power in patients with metabolic syndrome (22). In obese children and adolescents, it effectively identifies metabolic dysfunction-associated steatotic liver disease (MASLD) and IR (23, 24). Similarly, it has proven to be an accurate tool for identifying IR in Korean adults (25) and Mexican children (26). More importantly, the value of the SPISE index is not limited to cross-sectional associations; its longitudinal capability to predict future disease risk has also been preliminarily confirmed. For example, prospective studies have shown that a lower SPISE index predicts the risk of future cardiovascular events in adults with DM (27) and the development of glucose dysregulation in overweight or obese children (28). A study utilizing the CHARLS and ELSA cohorts further confirmed that a higher SPISE index is associated with a reduced risk of cardiovascular disease in middle-aged and elderly populations, highlighting its value as a cardiovascular risk assessment tool (29). Collectively, these lines of evidence suggest that the SPISE index is an effective indicator reflecting long-term metabolic health risks.

Although the SPISE index has demonstrated predictive value for the risk of cardiovascular disease and DM to some extent, existing research remains subject to certain limitations. On one hand, few prospective studies have specifically focused on its predictive value for IFG, a critical prediabetic state. On the other hand, the majority of current studies are based on Western populations or specific clinical cohorts. Given the known ethnic differences in body fat distribution and insulin sensitivity, the applicability of the SPISE index to the general Chinese population requires validation. Considering the high incidence of IFG in China and the fact that IFG represents a crucial window for preventive intervention, conducting a large-scale longitudinal study in Chinese adults with normal baseline glucose levels to precisely evaluate the predictive capacity of the SPISE index for new-onset IFG is of indispensable value for developing targeted and efficient primary prevention strategies. Therefore, this study aims to utilize a retrospective longitudinal cohort of the Chinese population to explore whether baseline SPISE index constitutes an independent risk factor for the future development of IFG, with statistical adjustments for covariates such as age, gender, blood pressure (BP), and family history. It is anticipated that the results of this study will provide critical evidence supporting the SPISE index as an early, simple, and low-cost screening tool for IFG risk in Chinese adults. This index holds promise as an effective means for identifying high-risk individuals and initiating early interventions in primary care settings and public health programs.

## Methods

### Data source

The data for this study were obtained from the Dryad Digital Repository, an internationally recognized open science platform that adheres to open access principles and curates publicly available research data across multidisciplinary fields. The dataset utilized in the present study is associated with a large-scale population-based cohort study of Chinese adults published in *BMJ Open* in 2018, titled “Association of body mass index and age with incident diabetes in Chinese adults: a population-based cohort study” (30). The raw data are accessible via the Digital Object Identifier associated with the original publication. This dataset encompasses longitudinal clinical follow-up records from participants in health screening programs spanning from 2010 to 2016. In accordance with the policies of the Dryad Digital Repository, secondary analysis of public data is permitted within ethical and legal frameworks to investigate novel scientific questions. Given that the original study received ethical approval from the Rich Healthcare Group in China, and the present secondary analysis involves no new participant intervention, neither additional ethical review nor re-acquisition of informed consent was required. All study procedures were conducted in strict adherence to the ethical guidelines stipulated in the Declaration of Helsinki and fully complied with relevant national laws, regulations, and institutional policies.

### Study population

**Figure 1:**
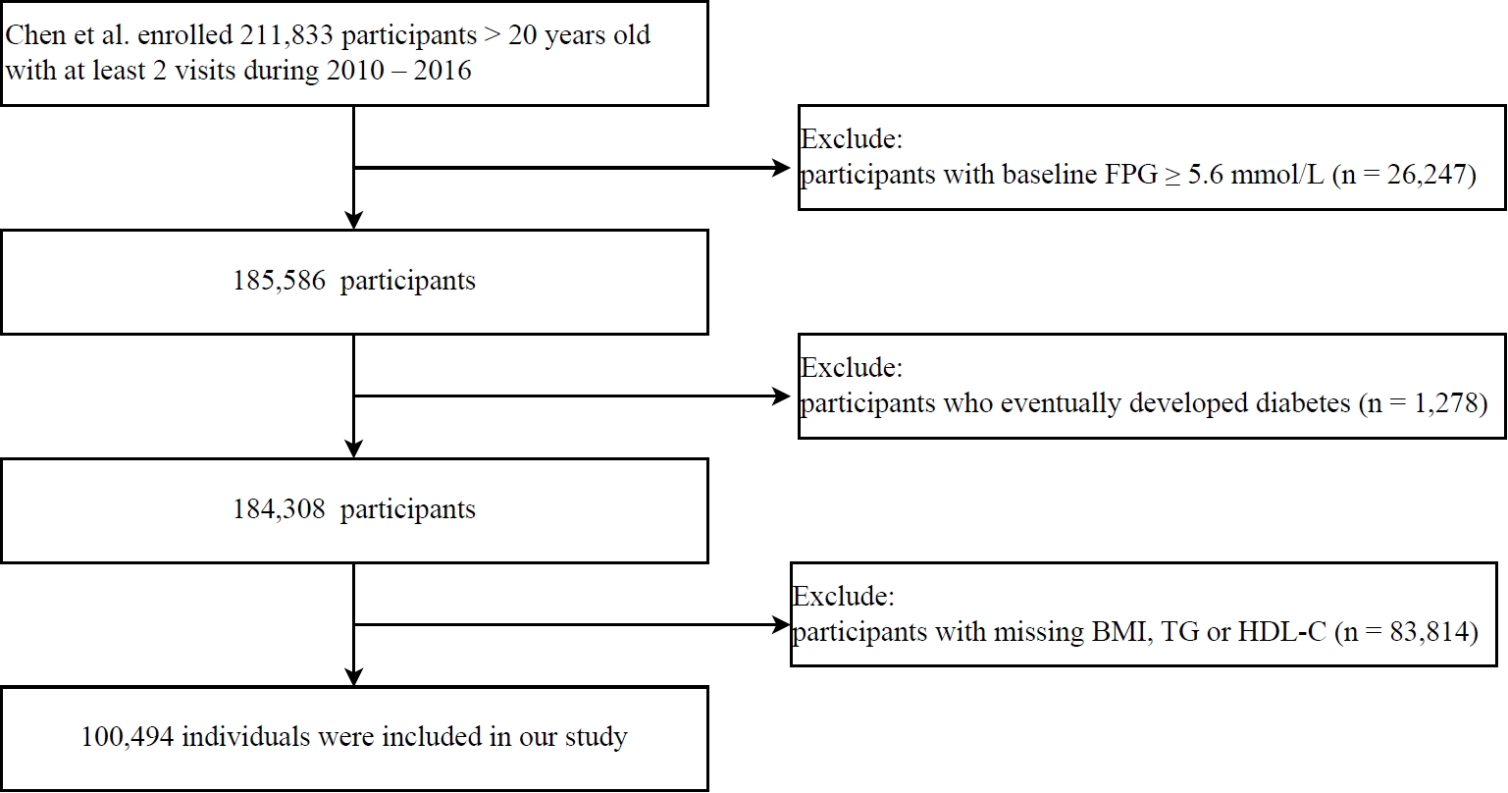
Flowchart of the present study.

This study is a secondary analysis based on a cohort from mainland China. Originally established by Chen et al., the cohort included 685,277 individuals. According to the predefined inclusion and exclusion criteria, 211,833 participants were initially selected as the baseline population for this analysis (24). The aim of the present study was to investigate the relationship between baseline SPISE index and the risk of developing IFG among individuals with normal baseline fasting glucose. Therefore, further screening was performed on the initial population: first, individuals with baseline fasting glucose ≥ 5.6 mmol/L were excluded; second, those diagnosed with DM during follow-up were removed; and finally, participants with missing data for key indicators such as BMI, HDL-C, or TG were excluded. After applying these criteria, a total of 100,494 participants were included in the final analysis. The detailed screening process of the study subjects is shown in **Figure 1**.

### Definitions of the exposure variable

The SPISE index was used as the primary exposure factor in this study, which is a continuous variable. It was estimated according to the formula proposed in the literature (30). The specific calculation formula is as follows: SPISE index = (600 * HDL-C [mg/dL]^0.185^)/(TG [mg/dL]^0.2^ * BMI [kg/m^2^]^1.338^).

### Definitions of the outcome variable

The primary outcome of this study was defined as incident IFG. According to the relevant guidelines of the American Diabetes Association (ADA), the diagnostic criterion for IFG is a measured FPG level between 5.6 mmol/L and 7.0 mmol/L in individuals without a history of diabetes. The follow-up time was calculated from the date of the baseline health assessment to the date of the last available test used for analysis.

### Other covariates

In this study, the following categories of covariates were included: (1) Demographic characteristics, including age and gender; (2) Anthropometric measurements, including height and body weight; (3) Blood biochemical indices, including FPG, total cholesterol (TC), TG, low-density lipoprotein cholesterol (LDL-C), HDL-C, alanine aminotransferase (ALT), aspartate aminotransferase (AST), blood urea nitrogen (BUN), and serum creatinine (Scr); (4) Behavioral and lifestyle factors, including smoking and drinking status; (5) Family history of diabetes.

In this study, a structured questionnaire was used to collect information on demographic characteristics, behavioral and lifestyle factors, and family disease history. Anthropometric measurements, including height, body weight, and BP, were taken by uniformly trained staff following standardized operating procedures. BMI was calculated as body weight (kg) divided by the square of height (m). Smoking and drinking status were categorized as “current,” “ever,” or “never” according to the classification criteria of the original database. Venous blood samples were collected from all participants after a fast of at least 10 hours by professional personnel. Relevant biochemical indices were measured using the Beckman Coulter AU5800 fully automated biochemical analysis system.

### Missing data processing

The dataset included in this analysis contained missing values for some variables. We addressed this issue following conventional strategies for handling missing data in observational epidemiological studies. Among the variables included in the analysis, a total of 10 variables had missing records, comprising 8 continuous variables and 2 categorical variables. The specific proportions of missing data are detailed in **Supplementary Table 1**. To reduce potential bias introduced by the missing data, we performed multiple imputations for the following continuous variables: ALT, AST, BUN, Scr, SBP, DBP, LDL-C, and TC. The procedure was implemented in the R environment with the MICE package (31). This method utilized multiple imputation by chained equations, performing 5 iterations to generate 5 complete datasets. It can effectively handle arbitrary missing data patterns and appropriately reflect the random components within the missing data, representing a currently robust statistical imputation approach.

For missing information in the two categorical variables, smoking and drinking status, the data were classified into a “Not recorded” category. To examine the reliability of the multiple imputations and verify the robustness of the findings, the primary regression analyses reported in the main text were based on the pooled dataset from the imputations. Furthermore, to comprehensively assess the stability of the conclusions, analyses based solely on the original complete data and on data after excluding individuals with missing covariates are presented simultaneously in the supplementary materials. The direction of association and the effect size between the SPISE index and the risk of incident IFG remained essentially consistent across the three data processing approaches, indicating that the main conclusions of this study are not sensitive to the choice of missing data handling method and the results demonstrated good stability.

### Statistical method

**Figure 2:**
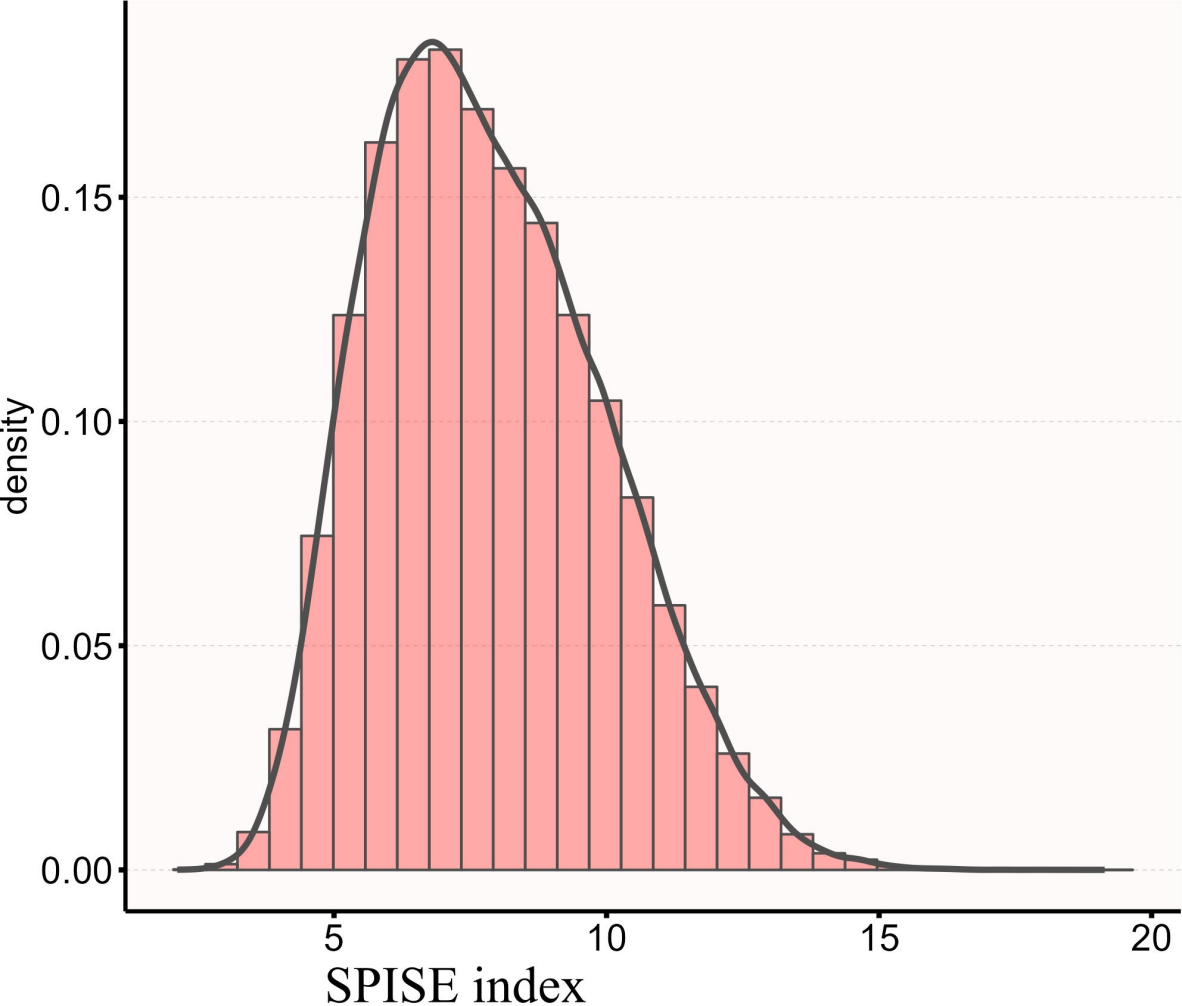
The distribution of SPISE index.

The SPISE index calculated in this study showed a normal distribution. The distribution of the SPISE index in the study population is shown in **Figure 2**. Using the quartiles of the SPISE index as cut-off points, all participants were divided into four groups: Q1 (≤ 6.243), Q2 (≤ 7.63), Q3 (≤ 9.283), and Q4 (≤ 19.138). The normality of continuous variables was assessed using the Kolmogorov-Smirnov test. Variables with a normal distribution are expressed as mean ± standard deviation, while non-normally distributed variables are described as median (interquartile range) [M (IQR)]. Categorical variables are presented as frequency (percentage) [n (%)]. For between-group comparisons, one-way analysis of variance was used for normally distributed continuous variables with homogeneity of variance; otherwise, the Kruskal-Wallis H test was applied. Comparisons between categorical variables were performed using the chi-square test.

To examine the relationship between different levels of the SPISE index and the risk of incident IFG, we plotted Kaplan-Meier (K-M) survival function curves corresponding to SPISE index quartiles. The significance of differences between groups was assessed using the log-rank test. Furthermore, multivariable Cox proportional hazards regression models were established to evaluate the association between the SPISE index and the risk of IFG development, calculating hazard ratios (HRs) and their 95% confidence intervals (CIs). To systematically examine the robustness of this association at different levels of confounding factor control, we constructed two hierarchically adjusted regression models: Model I was adjusted for age, gender, baseline FPG, SBP, and DBP; Model II (the fully adjusted model) further included ALT, AST, LDL-C, TC, BUN, Scr, smoking status, drinking status, and family history of diabetes at baseline based on Model I, to comprehensively control for the influence of potential confounders. To enhance the robustness of causal inference and to test the impact of different statistical models on the results, this study simultaneously constructed a multivariate logistic regression model to analyze the association between the SPISE index and the prevalence of IFG. The set of adjustment variables included was identical to that in the aforementioned Cox models. Concordant statistical conclusions from both types of models would significantly enhance the credibility of the study findings.

Furthermore, to thoroughly examine the linear or non-linear relationship between the SPISE index and the risk of IFG, a smooth curve was fitted using restricted cubic splines (RCS). Finally, subgroup analyses were performed, stratifying by variables including gender, age, BMI, systolic blood pressure (SBP), diastolic blood pressure (DBP), LDL-C, HDL-C, TC, TG, smoking status, drinking status, and family history of diabetes. Except for the stratification variable itself, Cox regression analyses within each subgroup were uniformly adjusted for the other covariates to assess the stability and consistency of the primary association across different demographic and clinical characteristics. Finally, AUROC analysis was performed to compare the predictive capability of the SPISE index and the triglyceride-glucose (TyG) index for incident IFG. The DeLong test was used to compare the area under the curves (AUCs).

### Statistical software

All results are reported in accordance with the STROBE statement. Statistical analyses were performed in the R environment (version 4.2.2; https://www.R-project.org/) and the Free Statistics Analysis Platform (version 2.1.1; https://www.clinicalscientists.cn/freestatistics). A two-tailed *P*-value of less than 0.05 was considered statistically significant. As the present study constitutes a secondary analysis of pre-existing data, no formal *a priori* sample size calculation was performed.

## Result

### Baseline characteristics of the study population

This study included a total of 100494 Chinese adults with normal FPG at baseline. During a median follow-up of 2.99 years, a total of 12,392 incident IFG cases were identified. The baseline characteristics of the overall population are presented in **Table 1**. The mean age was 42.92 ± 12.46 years, and 51.98% were male. The average height was 166.24 ± 8.30 cm, weight was 64.14 ± 11.91 kg, and BMI was 23.10 ± 3.22 kg/m^2^. The mean SPISE index was 7.85 ± 2.11. After grouping by SPISE index quartiles, all measured indices showed graded changes with increasing SPISE index. From Q1 to Q4, the age of the participants progressively decreased, the proportion of males decreased while that of females increased, and height, weight, and BMI all declined with higher SPISE index levels. SBP and DBP exhibited a decreasing trend. TG levels gradually decreased from Q1 to Q4, while HDL-C levels increased. TC and LDL-C decreased in parallel, and baseline FPG showed a slight decline with an increasing SPISE index. The median values for ALT and AST progressively decreased, and BUN and Scr also showed declining trends. The proportions of current and former smokers and drinkers were higher in the lower SPISE index groups and diminished with increasing SPISE index. All these changes were statistically significant (all *P* < 0.001), except for family history of diabetes (*P* = 0.266).

**Table 1:** Baseline characteristics of the study population by SPISE index quartiles.

Variables	Total (n = 100494)	SPISE index				
		Q1(n = 25124)	Q2 (n =25123)	Q3 (n =25123)	Q4 (n = 25124)	*P*-Value
Age (years), Mean ± SD	42.92 ± 12.46	45.96 ± 12.66	45.29 ± 12.82	42.29 ± 12.06	38.12 ± 10.60	< 0.001
Gender, n (%)						< 0.001
Male	52233 (51.98)	19537 (77.76)	15791 (62.85)	10869 (43.26)	6036 (24.02)	
Female	48261 (48.02)	5587 (22.24)	9332 (37.15)	14254 (56.74)	19088 (75.98)	
Height (cm), Mean ± SD	166.24 ± 8.30	168.76 ± 8.22	167.08 ± 8.48	165.24 ± 8.22	163.87 ± 7.39	< 0.001
Body weight (kg), Mean ± SD	64.14 ± 11.91	76.81 ± 10.15	67.09 ± 7.84	60.09 ± 6.93	52.56 ± 5.98	< 0.001
BMI (kg/m^2^), Mean ± SD	23.10 ± 3.22	26.91 ± 2.45	23.98 ± 1.52	21.95 ± 1.36	19.54 ± 1.43	< 0.001
SPISE index, Mean ± SD	7.85 ± 2.11	5.35 ± 0.65	6.93 ± 0.40	8.42 ± 0.48	10.71 ± 1.15	< 0.001
SBP (mmHg), Mean ± SD	118.08 ± 16.08	125.74 ± 16.02	120.70 ± 15.56	115.43 ± 14.90	110.44 ± 13.53	< 0.001
DBP (mmHg), Mean ± SD	73.76 ± 10.77	79.09 ± 10.97	75.17 ± 10.32	71.71 ± 9.83	69.06 ± 9.16	< 0.001
Baseline FPG (mmol/L), Mean ± SD	4.79 ± 0.47	4.87 ± 0.47	4.83 ± 0.47	4.77 ± 0.47	4.69 ± 0.47	< 0.001
TC (mmol/L), Mean ± SD	4.75 ± 0.88	5.03 ± 0.91	4.83 ± 0.88	4.64 ± 0.84	4.50 ± 0.80	< 0.001
TG (mmol/L), M (IQR)	1.06 (0.74, 1.58)	1.93 (1.44, 2.63)	1.23 (0.97, 1.60)	0.90 (0.70, 1.14)	0.66 (0.51, 0.84)	< 0.001
HDL-C (mmol/L), Mean ± SD	1.38 ± 0.30	1.20 ± 0.26	1.32 ± 0.25	1.43 ± 0.27	1.57 ± 0.31	< 0.001
LDL-C (mmol/L), Mean ± SD	2.74 ± 0.67	2.92 ± 0.70	2.84 ± 0.67	2.69 ± 0.64	2.53 ± 0.60	< 0.001
ALT (U/L), M (IQR)	17.70 (12.80, 26.60)	27.10 (19.00, 40.00)	19.80 (14.60, 27.70)	15.30 (11.90, 21.00)	13.00 (10.30, 17.00)	< 0.001
AST (U/L), M (IQR)	21.90 (18.30, 26.00)	24.60 (20.30, 30.30)	22.00 (19.00, 26.70)	21.00 (18.00, 24.95)	20.00 (17.20, 23.10)	< 0.001
BUN (mmol/L), Mean ± SD	4.63 ± 1.16	4.80 ± 1.14	4.73 ± 1.16	4.57 ± 1.17	4.42 ± 1.13	< 0.001
Scr (μmol/L), Mean ± SD	69.84 ± 15.71	76.11 ± 15.54	72.63 ± 15.27	67.88 ± 15.48	62.75 ± 13.13	< 0.001
Smoking status, n (%)						< 0.001
Current smoker	5363 (5.34)	2376 (9.46)	1482 (5.9)	961 (3.83)	544 (2.17)	
Ever smoker	1093 (1.09)	440 (1.75)	353 (1.41)	205 (0.82)	95 (0.38)	
Never smoker	21236 (21.13)	5121 (20.38)	5303 (21.11)	5364 (21.35)	5448 (21.68)	
Not recorded	72802 (72.44)	17187 (68.41)	17985 (71.59)	18593 (74.01)	19037 (75.77)	
Drinking status, n (%)						< 0.001
Current drinker	646 (0.64)	305 (1.21)	182 (0.72)	106 (0.42)	53 (0.21)	
Ever drinker	4559 (4.54)	1754 (6.98)	1345 (5.35)	948 (3.77)	512 (2.04)	
Never drinker	22487 (22.38)	5878 (23.4)	5611 (22.33)	5476 (21.8)	5522 (21.98)	
Not recorded	72802 (72.44)	17187 (68.41)	17985 (71.59)	18593 (74.01)	19037 (75.77)	
Family history of diabetes, n (%)						0.266
No	98280 (97.80)	24566 (97.78)	24584 (97.85)	24534 (97.66)	24596 (97.9)	
Yes	2214 (2.20)	558 (2.22)	539 (2.15)	589 (2.34)	528 (2.1)	
IFG, n (%)						< 0.001
No	88102 (87.67)	20238 (80.55)	21500 (85.58)	22694 (90.33)	23670 (94.21)	
Yes	12392 (12.33)	4886 (19.45)	3623 (14.42)	2429 (9.67)	1454 (5.79)	

Analysis by IFG outcome group, as shown in **Table 2**, revealed that the baseline characteristics of the study population who developed IFG exhibited trends similar to those of the low SPISE index group. Compared to the group that did not develop IFG, the IFG group was older, had a higher proportion of males, and exhibited higher levels of weight, BMI, SBP, DBP, FPG, TC, TG, LDL-C, ALT, AST, BUN, and Scr, while HDL-C and the SPISE index were lower. The proportion of current smokers was also higher in the IFG group. All aforementioned comparisons were statistically significant (all *P* < 0.001). The positive rate of family history of diabetes was not significantly different between the two groups (*P* = 0.011).

**Table 2:** Baseline characteristics of the study population by IFG outcome.

Variables	Non-IFG (n = 88102)	IFG (n =12392)	*P*-Value
Age (years), Mean ± SD	42.06 ± 12.04	49.01 ± 13.59	< 0.001
Gender, n (%)			< 0.001
Male	44469 (50.47)	7764 (62.65)	
Female	43633 (49.53)	4628 (37.35)	
Height (cm), Mean ± SD	166.17 ± 8.28	166.70 ± 8.43	< 0.001
Body weight (kg), Mean ± SD	63.59 ± 11.81	68.03 ± 11.91	< 0.001
BMI (kg/m^2^), Mean ± SD	22.91 ± 3.17	24.38 ± 3.25	< 0.001
SPISE index, Mean ± SD	7.98 ± 2.11	6.96 ± 1.84	< 0.001
SBP (mmHg), Mean ± SD	117.08 ± 15.62	125.17 ± 17.51	< 0.001
DBP (mmHg), Mean ± SD	73.22 ± 10.57	77.61 ± 11.37	< 0.001
Baseline FPG (mmol/L), Mean ± SD	4.75 ± 0.47	5.03 ± 0.40	< 0.001
TC (mmol/L), Mean ± SD	4.72 ± 0.88	4.92 ± 0.90	< 0.001
TG (mmol/L), M (IQR)	1.02 (0.72, 1.52)	1.30 (0.90, 1.95)	< 0.001
HDL-C (mmol/L), Mean ± SD	1.39 ± 0.31	1.34 ± 0.29	< 0.001
LDL-C (mmol/L), Mean ± SD	2.73 ± 0.67	2.84 ± 0.67	< 0.001
ALT (U/L), M (IQR)	17.10 (12.40, 26.00)	21.00 (14.70, 31.00)	< 0.001
AST (U/L), M (IQR)	21.60 (18.10, 26.00)	23.00 (19.00, 28.00)	< 0.001
BUN (mmol/L), Mean ± SD	4.60 ± 1.15	4.83 ± 1.17	< 0.001
Scr (μmol/L), Mean ± SD	69.38 ± 15.67	73.10 ± 15.64	< 0.001
Smoking status, n (%)			< 0.001
Current smoker	4519 (5.13)	844 (6.81)	
Ever smoker	937 (1.06)	156 (1.26)	
Never smoker	18903 (21.46)	2333 (18.83)	
Not recorded	63743 (72.35)	9059 (73.1)	
Drinking status, n (%)			< 0.001
Current drinker	545 (0.62)	101 (0.82)	
Ever drinker	3953 (4.49)	606 (4.89)	
Never drinker	19861 (22.54)	2626 (21.19)	
Not recorded	63743 (72.35)	9059 (73.1)	
Family history of diabetes, n (%)			0.011
No	86200 (97.84)	12080 (97.48)	
Yes	1902 (2.16)	312 (2.52)	

### Incidence of IFG across SPISE index quartile groups

**Figure 3:**
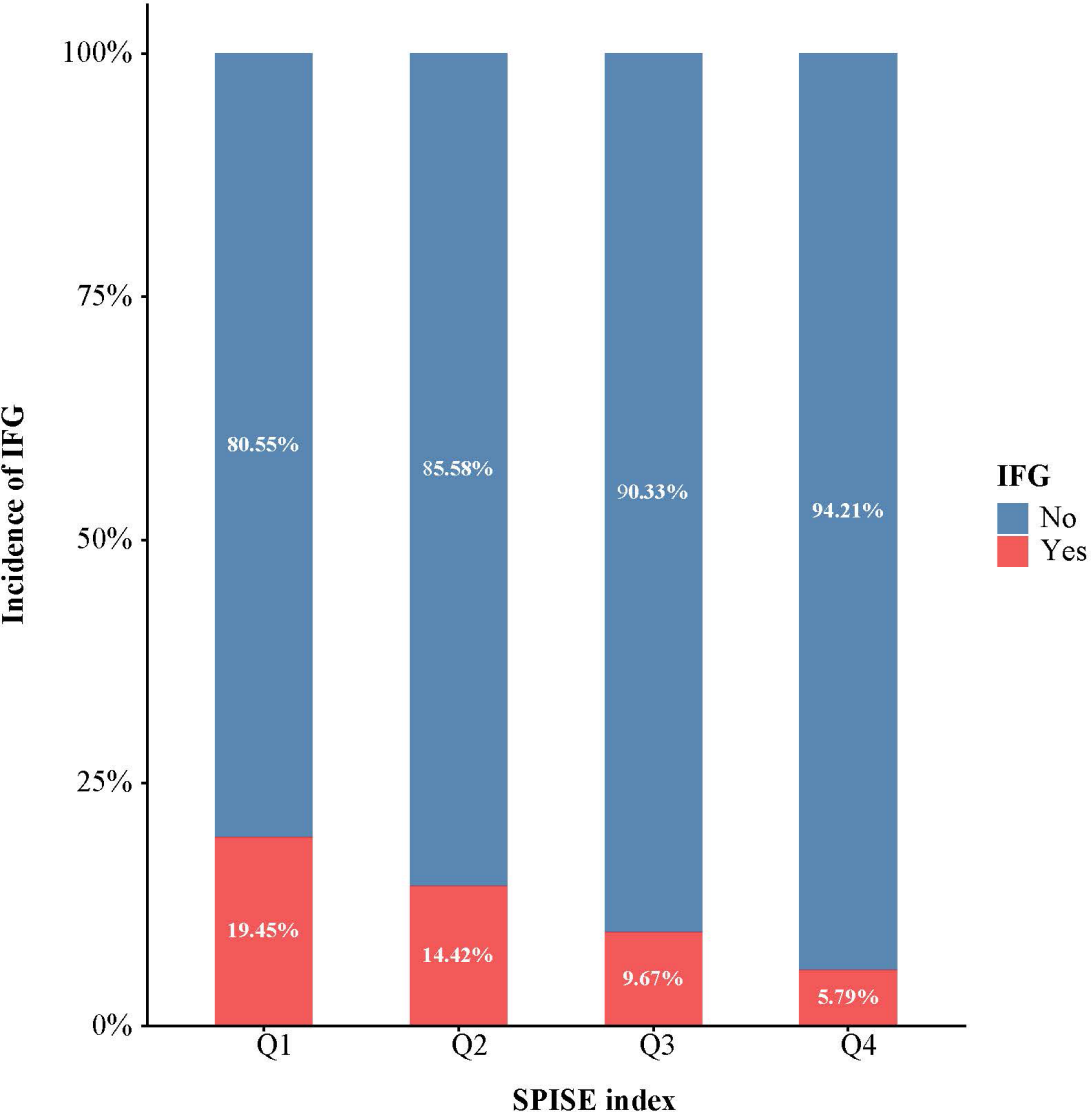
Stacked column chart of IFG incidence. The horizontal axis represents the SPISE index quartile groups, and the vertical axis depicts the incidence rate of IFG (%). Blue indicates participants who did not develop IFG, and red indicates those who did develop IFG.

By categorizing participants into SPISE index quartile groups, we observed a clear graded trend in the incidence of IFG. The stacked bar chart in **Figure 3** visually demonstrates a pronounced decreasing trend in IFG incidence with increasing SPISE index quartiles from Q1 to Q4. Specifically, the Q1 group exhibited the highest IFG incidence rate at 19.45%. As the SPISE index increased, the IFG incidence rate progressively declined: 14.42% in the Q2 group and further decreasing to 9.67% in the Q3 group. The lowest IFG incidence rate, at only 5.79%, was observed in the Q4 group. The overall IFG incidence rate in the study population was 12.33%. The differences in IFG incidence rates among the SPISE index quartile groups were statistically significant.

### Association analysis between SPISE index and IFG risk

This study employed Cox proportional hazards regression models and logistic regression models to assess the association between the SPISE index and the risk of incident IFG. The analytical results, covering models with different sets of adjusted variables, are presented in **Tables 3**, **4**.

**Table 3:** Association between SPISE index and risk of incident IFG using Cox proportional hazards regression in different models.

Variables	Crude model		Model I		Model II	
	HR (95%CI)	*P*-Value	HR (95%CI)	*P*-Value	HR (95%CI)	*P*-Value
SPISE index	0.80 (0.80, 0.81)	< 0.001	0.9 (0.89, 0.91)	< 0.001	0.91 (0.90, 0.92)	< 0.001
(SPISE index quartiles)						
Q1	1.00 (Reference)		1.00 (Reference)		1.00 (Reference)	
Q2	0.76 (0.73, 0.79)	< 0.001	0.90 (0.87, 0.94)	< 0.001	0.91 (0.87, 0.95)	< 0.001
Q3	0.51 (0.49, 0.54)	< 0.001	0.75 (0.71, 0.79)	< 0.001	0.77 (0.73, 0.81)	< 0.001
Q4	0.30 (0.28, 0.32)	< 0.001	0.58 (0.54, 0.62)	< 0.001	0.60 (0.56, 0.64)	< 0.001
*P* for trend		< 0.001		< 0.001		< 0.001

Crude model: we did not adjust other covariates.

Model I: adjusted for age, gender, FPG, SBP, and DBP at baseline.

Model II: further adjusted for ALT, AST, LDL-C, TC, BUN, Scr, smoking status, drinking status, and family history of diabetes at baseline.

**Table 4:** Association between SPISE index and incident IFG, assessed using​ logistic​ regression in different models.

Variables	Crude model		Model I		Model II	
	OR (95%CI)	*P*-Value	OR (95%CI)	*P*-Value	OR (95%CI)	*P*-Value
SPISE index	0.77 (0.76, 0.78)	< 0.001	0.87 (0.86, 0.88)	< 0.001	0.88 (0.87, 0.9)	< 0.001
(SPISE index quartiles)						
Q1	1.00 (Reference)		1.00 (Reference)		1.00 (Reference)	
Q2	0.70 (0.67, 0.73)	< 0.001	0.81 (0.77, 0.85)	< 0.001	0.85 (0.81, 0.90)	< 0.001
Q3	0.44 (0.42, 0.47)	< 0.001	0.65 (0.61, 0.69)	< 0.001	0.69 (0.65, 0.74)	< 0.001
Q4	0.25 (0.24, 0.27)	< 0.001	0.50 (0.47, 0.54)	< 0.001	0.54 (0.50, 0.58)	< 0.001
*P* for trend		< 0.001		< 0.001		< 0.001

Crude model: we did not adjust other covariates.

Model I: adjusted for age, gender, FPG, SBP, and DBP at baseline.

Model II: further adjusted for ALT, AST, LDL-C, TC, BUN, Scr, smoking status, drinking status, and family history of diabetes at baseline.

In the crude model, for each one-unit increase in the SPISE index, the risk of IFG incidence was significantly reduced, with an HR of 0.8 and a 95% CI of 0.8 to 0.81. Analysis by SPISE index quartile groups, using Q1 as the reference, showed progressively decreasing HRs for Q2, Q3, and Q4: 0.76, 0.51, and 0.3, respectively. All comparisons were statistically significant. A trend test indicated a significant decreasing trend in IFG risk with increasing SPISE index quartiles.

In Model I, after adjusting for age, gender, baseline FPG, SBP, and DBP, the HR per one-unit increase in SPISE index was 0.9. The quartile analysis yielded HRs of 0.9, 0.75, and 0.58 for Q2, Q3, and Q4, respectively, compared to Q1. All results remained significant, with a significant trend.

In Model II, with further adjustment for ALT, AST, LDL-C, TC, BUN, Scr, smoking status, drinking status, and family history of diabetes, the HR per one-unit increase in SPISE index was 0.91. The corresponding HRs for the quartile groups were 0.91, 0.77, and 0.6 for Q2, Q3, and Q4, respectively. All associations and the trend test remained statistically significant.

The results from the logistic regression models were consistent with those from the Cox models. In the crude logistic model, the odds ratio (OR) per one-unit increase in the SPISE index was 0.77. The ORs for the quartile groups were 0.70, 0.44, and 0.25 for Q2, Q3, and Q4, respectively, with a significant trend. In Model I, the adjusted OR per unit increase was 0.87, and the quartile ORs were 0.81, 0.65, and 0.50. In the fully adjusted Model II, the OR per unit increase was 0.88, and the quartile ORs were 0.85, 0.69, and 0.54. All results and trend tests were statistically significant. The SPISE index was inversely associated with the risk of IFG, and this association remained consistent across different adjustment models, supporting the SPISE index as a potential predictor for IFG risk.

### Survival analysis

**Figure 4:**
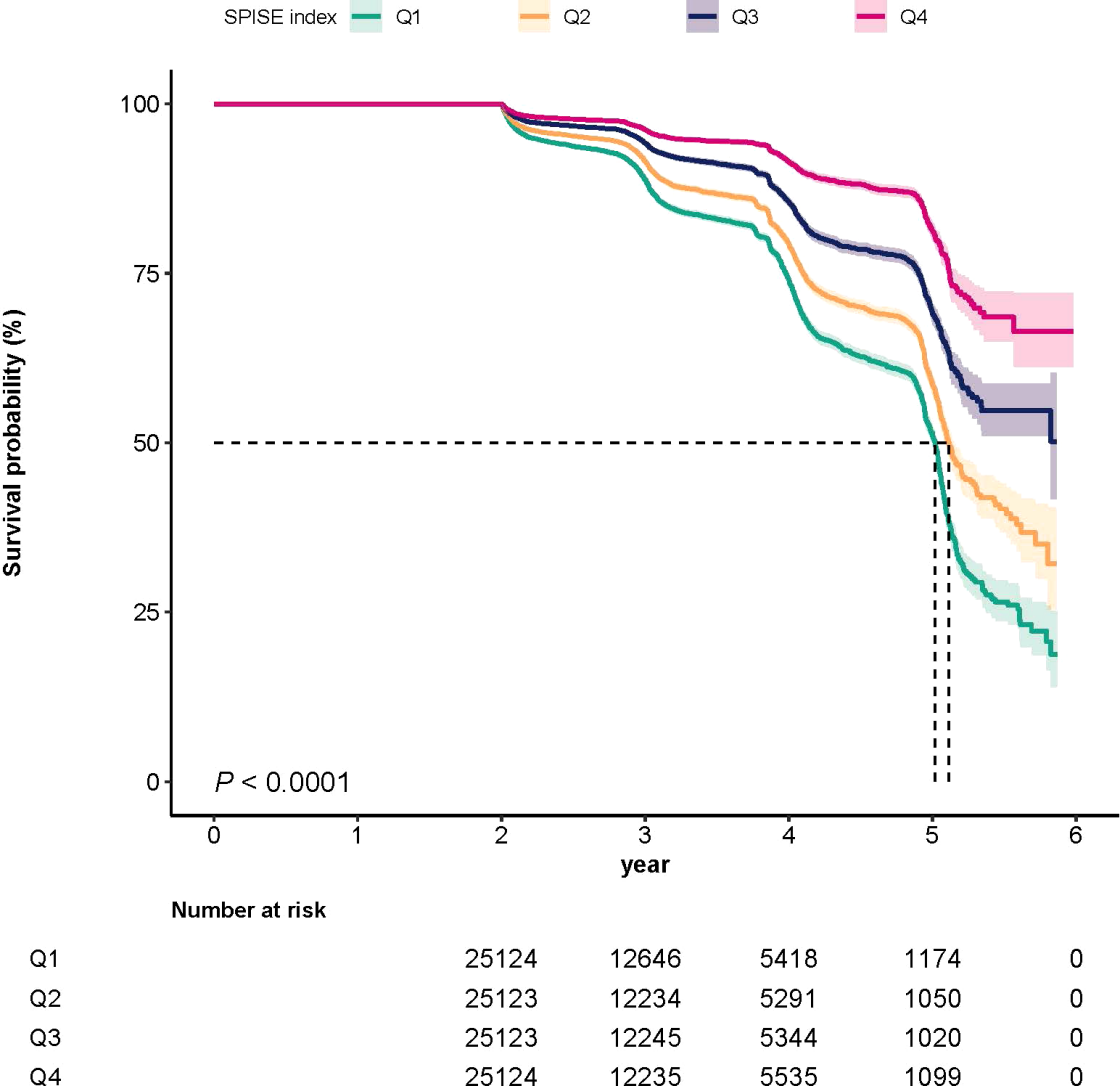
K-M curves illustrate incident IFG risk by SPISE index quartiles. A pronounced divergence in survival curves was observed among the SPISE index quartile groups (Q1-Q4), with statistical significance confirmed by a log-rank test (*P* < 0.0001).

This study utilized K-M curves to assess the impact of SPISE index quartile groups on the risk of incident IFG. The survival analysis results presented in **Figure 4** demonstrated significant differences in the IFG survival curves among the different SPISE index quartile groups (log-rank test *P* < 0.0001). The survival curve for the Q1 group was positioned lowest, indicating the highest risk of IFG. As the SPISE index quartile increased, the survival curves progressively shifted upward, with the Q4 group exhibiting the highest curve position, suggesting the lowest IFG risk. These findings support a clear inverse relationship between the SPISE index and the risk of developing IFG, with this association showing a consistent graded change across the groups.

### Linear relationship between SPISE index and IFG risk

**Figure 5:**
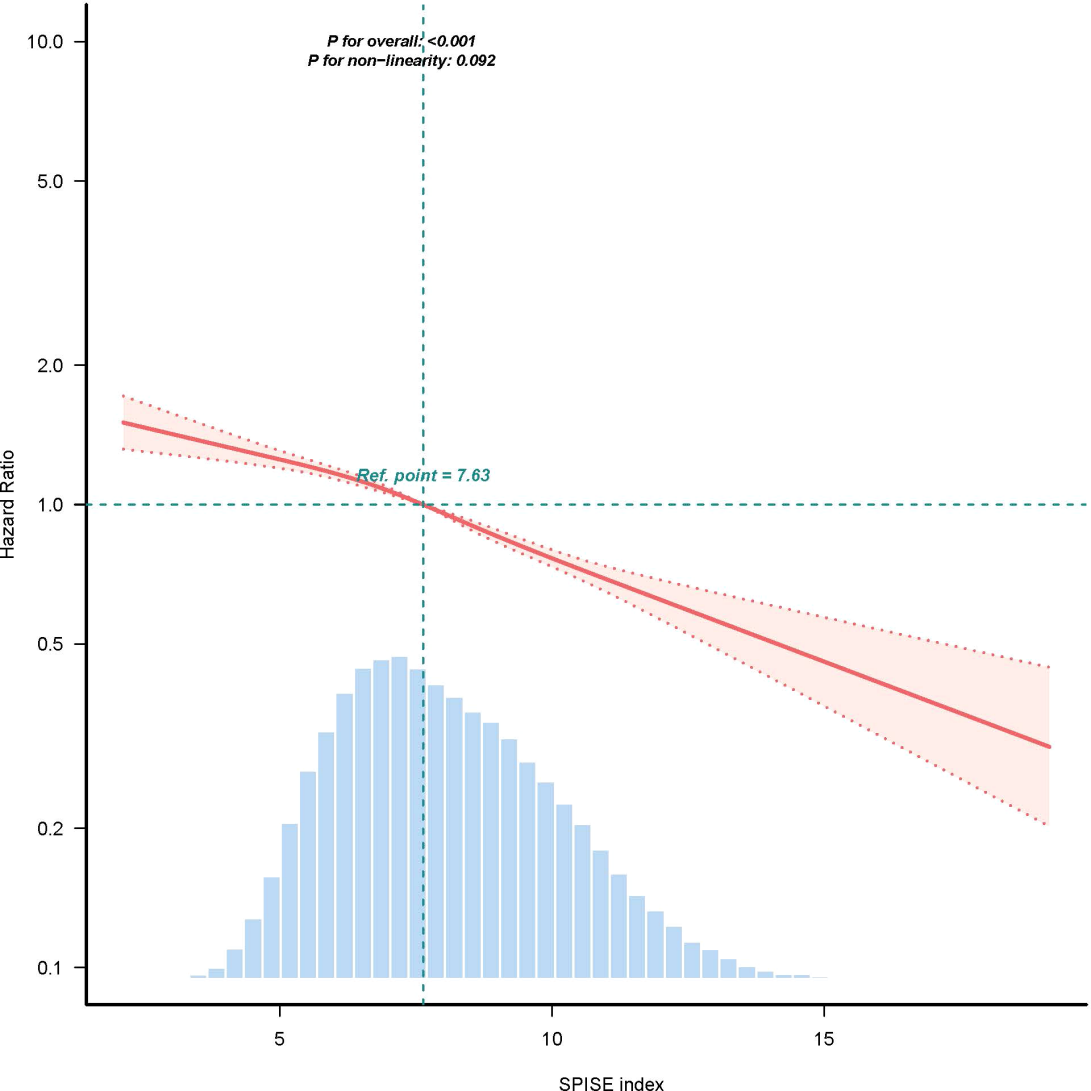
The linear relationship between SPISE index and risk of incident IFG. We adjusted age, gender, FPG, SBP, DBP, ALT, AST, LDL-C, TC, BUN, Scr, smoking status, and family history of diabetes at baseline.

This study assessed the linear relationship between the SPISE index and the risk of incident IFG using RCS analysis. As shown in **Figure 5**, conducted after adjusting for covariates including age, gender, FPG, SBP, DBP, ALT, AST, LDL-C, TC, BUN, Scr, smoking status, drinking status, and family history of diabetes, the analysis demonstrated a gradual decrease in IFG risk with increasing SPISE index. This dose-response relationship further supports the inverse correlation between SPISE index as a continuous variable and the risk of IFG.

### Subgroup analysis

**Figure 6:**
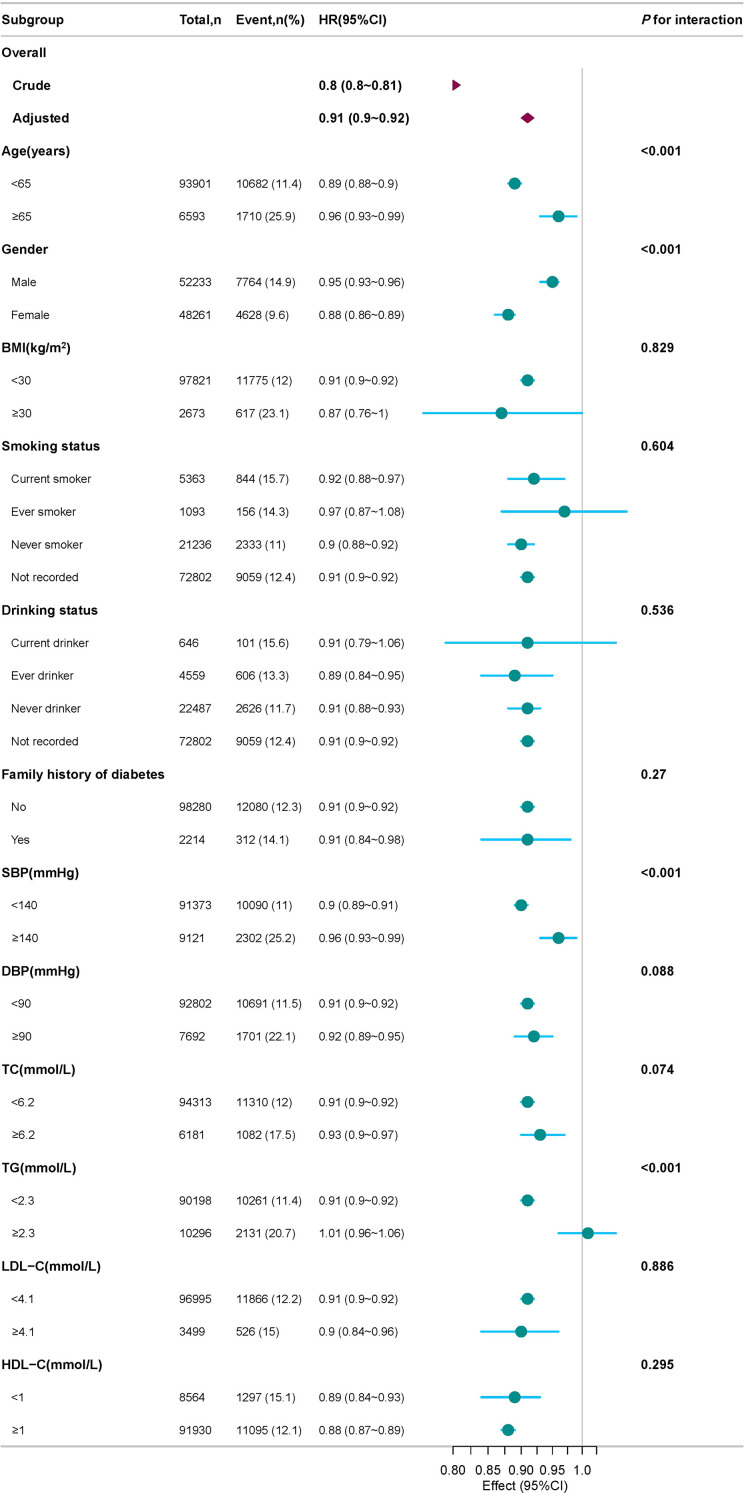
Forest plot of subgroup analysis of the association between SPISE index and risk of incident IFG.

This study evaluated the consistency of the association between the SPISE index and IFG risk across different population characteristics through subgroup analyses. As shown in the forest plot in **Figure 6**, the results are presented stratified by key variables, including age, gender, BMI, SBP, DBP, TG, TC, HDL-C, LDL-C, smoking status, drinking status, and family history of diabetes. The analyses across all subgroups demonstrated that the inverse association between SPISE index and IFG risk remained stable. The subgroup analysis results further confirmed the robustness of the association between the SPISE index and IFG risk, suggesting that this index has good predictive value across populations with different characteristics.

### Sensitivity analysis

In the analysis of the original dataset, the Cox regression model showed a HR of 0.89 per one-unit increase in the SPISE index, with a 95% CI of 0.86 to 0.93. The logistic regression analysis yielded an OR of 0.85, with a 95% CI of 0.82 to 0.89. Both results remained statistically significant. While the CIs for some quartile groups widened and the *P* values approached borderline significance, the overall trend was consistent with the primary analysis. The analysis after excluding incomplete data showed that the HR for SPISE index in the Cox regression was 0.89, and the OR in the logistic regression was 0.85. These results were highly concordant with those from the original dataset analysis. Although the reduction in sample size led to further widening of CIs for some quartile groups, the magnitude of risk reduction in the highest quartile group remained significant, and the trend tests were all statistically significant. **Supplementary Figures 1**, **2** further explore the linear relationship between SPISE index and IFG risk. The results from both figures indicate a linear inverse association between the SPISE index and the risk of incident IFG.

### Predictive performance of SPISE index versus TyG index

**Figure 7:**
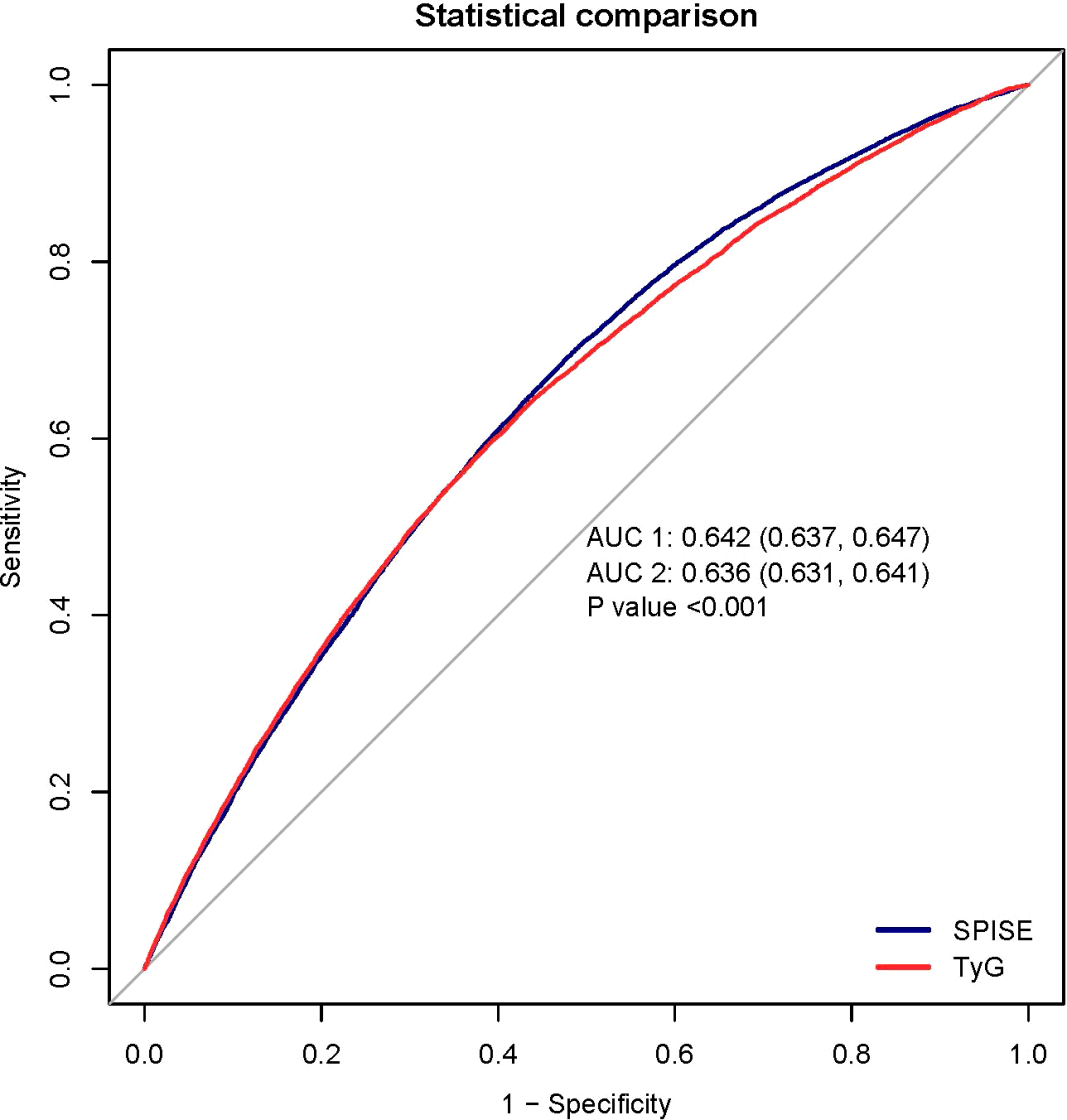
Comparison of the predictive performance between the SPISE index and TyG index for incident IFG.

To further evaluate the clinical utility of the SPISE index, we compared its predictive performance with the TyG index using AUROC curve analysis. The results demonstrated that the AUC for the SPISE index was 0.642 (95% CI: 0.637-0.647), which was significantly higher than that of the TyG index (AUC: 0.636, 95% CI: 0.631-0.641; *P* < 0.001) (**Figure 7**). This indicates a slightly but significantly better discriminative ability of the SPISE index for incident IFG in this population.

## Discussion

Through a secondary analysis of a large Chinese community-based cohort, this study systematically evaluated, for the first time, the longitudinal association between the baseline SPISE index and the future risk of incident IFG. The core finding is that among 100,494 Chinese adults with normal FPG at baseline, a higher baseline SPISE index was independently associated with a significantly reduced risk of developing IFG, demonstrating a clear dose-response relationship. In the fully adjusted Cox proportional hazards model, which controlled for a series of potential confounders including age, gender, baseline blood glucose, SBP, DBP, TC, LDL-C, AST, ALT, BUN, Scr, smoking and drinking status, and family history of diabetes, each one-unit increase in the SPISE index corresponded to an approximately 9% reduction in IFG risk. When analyzed by SPISE index quartiles, individuals in the highest quartile had approximately a 40% lower risk of developing IFG compared to those in the lowest quartile. Multivariable logistic regression analysis yielded highly consistent results. RCS analysis revealed a continuous, linear inverse relationship between SPISE index and IFG risk. Furthermore, extensive subgroup analyses (stratified by age, gender, BMI, SBP, DBP, TC, TG, LDL-C, HDL-C, smoking status, drinking status, and family history of diabetes) and sensitivity analyses (including analyses based on the original data and after excluding cases with missing values) confirmed the robustness and consistency of this association. These results collectively suggest that the SPISE index, as a simply calculated composite measure, may be a potentially effective tool for predicting the risk of IFG onset in the Chinese adult population. By employing a series of rigorous and scientific analytical strategies, this study strengthens the understanding of the association between the SPISE index and IFG risk in Chinese adults. The findings indicate that the SPISE index, a simple composite metric integrating HDL-C, TG, and BMI, can comprehensively reflect an individual’s insulin sensitivity and metabolic health status. Its predictive power has been strongly validated for the specific outcome of IFG, offering a promising quantitative tool for large-scale prediabetes risk screening in resource-limited settings.

The SPISE index is a novel composite metric integrating HDL-C, TG, and BMI (21). The significant inverse association observed in this study between the SPISE index and the risk of incident IFG is biologically plausible. A higher SPISE index directly reflects a more favorable lipid profile and lower adiposity, both of which are key factors for maintaining normal insulin sensitivity. From a pathophysiological perspective, low HDL-C and high TG levels are central features of atherogenic dyslipidemia, which is intricately intertwined with an insulin-resistant state. This dyslipidemia may impair insulin signaling through pathways such as affecting lipid metabolism, promoting inflammation, and causing endothelial dysfunction (32, 33). Concurrently, elevated BMI, a simple indicator of obesity, is particularly associated with visceral fat accumulation. Excessive visceral adipose tissue is not an inert energy storage organ but an active endocrine organ secreting substantial amounts of free fatty acids, adipokines (such as leptin and adiponectin), and pro-inflammatory cytokines (such as tumor necrosis factor-α and interleukin-6). The abnormal secretion of these factors can induce systemic chronic low-grade inflammation and oxidative stress, directly interfering with insulin receptor substrate-1 and phosphatidylinositol 3-kinase signaling pathways in skeletal muscle, liver, and adipose tissue, ultimately leading to reduced insulin sensitivity (34–36). IR is a core driver in the development of DM and its prediabetic stage, IFG. When insulin sensitivity declines, the regulation of glucose homeostasis in multiple organs is compromised: insulin-stimulated glucose uptake and utilization are significantly reduced in skeletal muscle; insulin’s suppression of hepatic gluconeogenesis is weakened, leading to inappropriately increased hepatic glucose output; and insulin’s inhibition of lipolysis in adipose tissue is impaired, elevating circulating free fatty acid levels. These, in turn, exacerbate hepatic and muscular IR and promote hepatic gluconeogenesis (37, 38). The combined effect of these pathophysiological changes ultimately leads to a progressive increase in FPG levels until reaching the diagnostic threshold for IFG. In the present study, a lower SPISE index serves as a hallmark of IR and metabolic dysfunction, and its association with a higher IFG risk aligns with this mechanism. This finding is highly consistent with conclusions from several recent studies utilizing the SPISE index to assess various health outcomes, collectively supporting its validity as a surrogate marker of insulin sensitivity. For instance, the SPISE index was proven to be an accurate tool for identifying IR in Mexican children (26). Among Korean adults, a higher SPISE index was associated with a lower risk of periodontitis (39), a chronic inflammatory disease whose pathogenesis is closely linked to systemic IR. More importantly, a prospective cohort study involving middle-aged and elderly populations in China and the UK confirmed that a higher SPISE index predicts a lower risk of incident cardiovascular disease (29). These studies, from different perspectives including IR, inflammatory status, and long-term cardiovascular outcomes, corroborate the link between the metabolic health status reflected by the SPISE index and the risk of various diseases. Therefore, by extending the predictive value of the SPISE index to IFG—a critical “sentinel” event in the development of DM—this study not only further establishes its role in the early identification of glucose metabolism abnormalities but also provides strong supporting evidence for the underlying biological rationale: assessing insulin sensitivity to predict the risk of disrupted glucose homeostasis.

The results of this study are highly consistent with the majority of recent evidence regarding the association between the SPISE index and the risk of various metabolic disorders and cardiovascular diseases, and for the first time explicitly extend its predictive value to the specific outcome of incident IFG in a Chinese adult population. Existing research has clearly established the SPISE index as an effective tool for identifying IR and metabolic syndrome. For instance, a cross-sectional study of Mexican children and adolescents demonstrated the excellent discriminatory ability of the SPISE index for identifying IR (26). Another cross-sectional analysis of children and adolescents from the Korea National Health and Nutrition Examination Survey also found a significant inverse association between the SPISE index and the risk of metabolic syndrome, with its predictive performance superior to commonly used markers such as HOMA-IR, METS-IR, TyG, and TG/HDL ratio (40). This cross-sectional evidence provides a theoretical basis for using the SPISE index as a baseline surrogate marker of insulin sensitivity for prospective risk prediction in the current study. Recent prospective cohort studies have revealed the predictive value of the SPISE index for long-term health outcomes, aligning with the longitudinal design of this investigation. A prospective cohort study involving middle-aged and elderly populations in China and the UK confirmed that a higher baseline SPISE index significantly predicted a lower risk of incident cardiovascular disease (29). While these studies advanced the application of the SPISE index from cross-sectional association to longitudinal risk prediction, their focus remained primarily on established endpoints like cardiovascular disease or diabetic complications. A key highlight of the present study is that it is the first to systematically evaluate the association between the SPISE index and incident IFG—a critical precursor stage to DM—in a large-scale community-based cohort of Chinese adults. Although previous studies explored the relationship between SPISE index and composite conditions like metabolic syndrome, research specifically targeting IFG, an important stage for DM prevention, was lacking. The findings of this study fill this gap and significantly advance the predictive window of the SPISE index, enabling its use to identify individuals who, while not yet meeting the diagnostic criteria for metabolic syndrome, are already at an early stage of risk for glucose metabolism abnormalities. Furthermore, the effect strength observed in this study is comparable to that reported in the aforementioned prospective study involving cardiovascular outcomes (29), further supporting the potential of the SPISE index as a robust risk prediction marker across the continuous disease spectrum from “IR to prediabetes to cardiovascular disease.” This study confirms that the SPISE index, a simple and inexpensive index, can be used not only to assess current metabolic status but also to effectively predict the early risk of future deterioration in glucose metabolism. This provides a powerful tool for the primary prevention and early screening of prediabetes in broader populations.

The findings of this present study hold clear value for clinical practice and public health intervention. The SPISE index, a simple surrogate marker calculable using only routine lipid profiles and BMI, provides a highly cost-effective tool for identifying individuals at high risk for DM in resource-limited primary care settings and large-scale community screenings. Compared to traditional indices like HOMA-IR, which requires the measurement of fasting insulin and glucose, the SPISE index avoids the need for expensive assays and invasive procedures, making it more feasible for implementation in primary care scenarios (41). This study confirms that a lower SPISE index can effectively predict the future risk of incident IFG even among a general population with normal baseline glucose levels. This suggests that clinicians and public health practitioners could incorporate the SPISE index into routine health check-ups or cardiovascular metabolic risk assessment screenings to identify “high-risk” individuals who, despite not yet exhibiting overt glucose abnormalities, already harbor underlying IR and metabolic dysfunction. At the public health level, these results offer a new perspective for developing targeted prevention strategies for prediabetes. Utilizing the SPISE index for risk stratification enables public health programs to more effectively identify and prioritize the highest-risk individuals for intervention, allocating limited resources efficiently to implement primary prevention measures focused on lifestyle modification, thereby delaying or preventing progression to DM. Furthermore, the parameters used to calculate the SPISE index are themselves modifiable metabolic markers, providing tangible biological indicators for monitoring intervention efficacy (42). For example, improvements in these parameters through weight loss, dietary adjustments, and increased physical activity should theoretically be accompanied by an increase in the SPISE index, which may translate into a reduced future DM risk. The findings also support the integration of metabolic health assessment into broader health management contexts. For instance, in the field of occupational health, existing research suggests that psychosocial factors such as low social support are associated with a higher DM risk (43). Therefore, within workplace health promotion programs, incorporating simple metabolic risk assessments using indices like the SPISE index, alongside traditional health education and fostering supportive social environments, could form part of a comprehensive strategy for preventing chronic diseases in the working population. In summary, promoting the use of the SPISE index can contribute to shifting the focus of DM management “downstream” to primary care and “upstream” to early prevention, moving from a disease-treatment model to a risk-prevention paradigm. This has long-term significance for reducing both individual disease burden and pressure on public health systems.

This study possesses several important methodological strengths. First, it employed a retrospective longitudinal cohort design. This design allowed for the assessment of the exposure at baseline and the prospective observation of its association with future incident IFG events, thereby providing stronger temporal sequence evidence for the SPISE index as a predictive biomarker and reducing the possibility of reverse causality common in cross-sectional studies. Second, the study focused on a well-defined and clinically significant early endpoint of glucose metabolism abnormality: incident IFG. While prospective studies have confirmed that the SPISE index can predict long-term outcomes such as cardiovascular disease (29), prospective evidence specifically targeting IFG, a key event in the development of DM, was lacking. This study fills this gap, positioning the predictive value of the SPISE index at an earlier stage of the disease continuum, which is crucial for implementing prevention-oriented public health strategies. Third, the study included a community-based Chinese adult population with normal FPG at baseline. This inclusion criterion ensured that all participants were free of IFG or DM at the outset, enabling a clear evaluation of the predictive ability of the SPISE index for the risk of incident IFG, avoiding the potential influence of existing disease status on baseline metabolic indicators. Furthermore, data based on a large-scale community cohort generally have good external validity, meaning the findings may be more broadly applicable to the general adult population in China. Finally, the statistical analysis concurrently employed both multivariable Cox proportional hazards models and logistic regression models, with systematic adjustment for a series of potential confounding factors, including demographic characteristics, lifestyle factors, and baseline metabolic indices. This rigorous statistical approach helps to more accurately estimate the independent association between the SPISE index and IFG risk, enhancing the credibility of the findings.

Despite the aforementioned strengths, several limitations of this present study should be acknowledged. First, this is an observational study. Although a longitudinal cohort design was employed, establishing a temporal sequence, the possibility of residual confounding or reverse causality cannot be completely ruled out. For instance, certain unmeasured factors such as genetic predisposition, detailed dietary and physical activity patterns, or environmental exposures might simultaneously influence both the SPISE index and the risk of IFG, and such factors were not systematically collected and adjusted for in this study. Second, the SPISE index, as a surrogate marker of insulin sensitivity, inherently carries some degree of measurement error and biological variability. This index relies on three parameters: BMI, fasting TG, and HDL-C. BMI does not distinguish between fat and muscle mass, while TG and HDL-C levels are influenced by recent diet, physical activity, and intra-individual biological fluctuations. Although standardized measurements were used, a single measurement may not fully represent an individual’s long-term metabolic status. Furthermore, the SPISE index primarily reflects hepatic and adipose tissue insulin sensitivity, and its assessment of skeletal muscle insulin sensitivity may be relatively limited. Third, the primary endpoint of this study was incident IFG, defined based on a single FPG measurement. Although this follows internationally accepted diagnostic criteria, a single measurement can be affected by an individual’s short-term physiological state, posing a risk of measurement error and potential misclassification. Future studies incorporating confirmatory tests such as the oral glucose tolerance test or hemoglobin A1c for endpoint determination would improve the accuracy of outcome definition. Fourth, the lack of a physiological gold standard, such as the hyperinsulinemic-euglycemic clamp, is a limitation that restricts our ability to perform direct validation. Consequently, our findings should be interpreted as an indirect validation of the SPISE index as a surrogate marker for insulin sensitivity in a large-scale population. Similarly, the lack of fasting insulin data in this secondary screening cohort precluded a direct comparison between the SPISE index and HOMA-IR. Finally, the study population consisted of Chinese community-dwelling adults. While this enhances the applicability of the results within the Chinese population, it may limit the generalizability of the findings. The association between the SPISE index and IFG risk might differ in populations of other races, ethnicities, or with specific metabolic characteristics, such as those with diagnosed MASLD. Therefore, caution is warranted when extrapolating the conclusions of this study to other populations.

Based on the findings, strengths, and limitations of the present study, future research could explore the following directions to further refine the application value of the SPISE index in the early prevention of DM. First, external validation in populations of different races and ethnicities is needed to determine the applicability of the SPISE index. When conditions permit, it will be necessary to conduct prospective cohort studies in other major racial groups, such as African, European, and South Asian populations, to validate the predictive ability of the SPISE index for incident IFG and to establish population-specific risk stratification cut-off points. This is crucial for its precise application on a global scale. Second, exploring the association between the SPISE index and a broader range of metabolic diseases, as well as the underlying mechanisms. While this study confirmed the association between the SPISE index and IFG, future research could extend its predictive value to the early risk assessment of complications such as DM, non-alcoholic fatty liver disease (NAFLD), and cardiovascular disease. Concurrently, integrating omics technologies (e.g., metabolomics, proteomics) to investigate the molecular signatures underlying a low SPISE index could reveal its links to specific pathophysiological mechanisms, such as adipose tissue dysfunction and chronic low-grade inflammation. Third, integrating psychosocial and behavioral factors to construct a multidimensional risk prediction model. A limitation of this study was the lack of systematic measurement of psychosocial factors. Growing evidence indicates that psychosocial factors such as social support, work stress, depression, and anxiety are independent influencers of IR and DM risk. Future studies could incorporate standardized psychosocial assessment tools alongside the collection of biomarkers like the SPISE index to investigate whether these factors modify or mediate the association between the SPISE index and IFG risk. By integrating data from biomarkers, psychosocial factors, and health behaviors, it may be possible to build a more effective and comprehensive risk assessment model, providing a basis for developing personalized, integrated intervention strategies. Fourth, designing interventional studies to verify the value of the SPISE index as a dynamic monitoring indicator. Ultimately, the clinical value of the SPISE index lies not only in risk identification but also in the dynamic monitoring of intervention effects. There is a need to design randomized controlled trials or pragmatic clinical trials to assess whether intensive lifestyle interventions or pharmacological interventions aimed at improving the SPISE index can effectively reduce the risk of incident IFG or DM in high-risk populations. Such research can directly test whether improving the metabolic health status represented by the SPISE index constitutes an effective disease prevention strategy, thereby providing the highest level of evidence for transforming the SPISE index from a predictive tool into an interventional target.

## Conclusion

This retrospective longitudinal cohort analysis demonstrated that among community-dwelling Chinese adults with normal baseline blood glucose, a lower SPISE index was independently and inversely associated with an increased risk of future incident IFG. Our findings suggest that the SPISE index could potentially be implemented in routine clinical practice as a simple and cost-effective tool for identifying individuals at high risk for IFG, facilitating the early identification and risk stratification of DM.

## Data Availability

Publicly available datasets were analyzed in this study. The dataset presented in this study is available in the online repository (https://doi.org/10.5061/dryad.ft8750v).

## Glossary

IR: Insulin resistance
IFG: Impaired fasting glucose
DM: Diabetes mellitus
OGTT: Oral glucose tolerance tests
HOMA-IR: Homeostatic Model Assessment for Insulin Resistance
SPISE: Single Point Insulin Sensitivity Estimator
HDL-C: High-density lipoprotein cholesterol
TG: Triglyceride
BMI: Body mass index
AUROC: Area under the receiver operating characteristic curve
MASLD: Metabolic dysfunction-associated steatotic liver disease
CHARLS: China Health and Retirement Longitudinal Study
ELSA: English Longitudinal Study of Ageing
BP: Blood pressure
ADA: American Diabetes Association
FPG: Fasting plasma glucose
TC: Total cholesterol
LDL-C: Low-density lipoprotein cholesterol
ALT: Alanine aminotransferase
AST: Aspartate aminotransferase
BUN: Blood urea nitrogen
Scr: Serum creatinine
SBP: Systolic blood pressure
DBP: Diastolic blood pressure
MICE: Multivariate Imputation by Chained Equations
IQR: Interquartile range
K-M: Kaplan-Meier
HR: Hazard ratio
CI: Confidence interval
OR: Odds ratio
RCS: Restricted cubic splines
STROBE: Strengthening the Reporting of Observational Studies in Epidemiology
QUICKI: Quantitative Insulin Sensitivity Check Index
NAFLD: Non-alcoholic fatty liver disease.
