# Supplementary material for: Single point insulin sensitivity estimator index and incident impaired fasting glucose in Chinese adults: a retrospective cohort study
Source: Front Endocrinol (Lausanne). 2026 Jun 5;17:1810066. doi: 10.3389/fendo.2026.1810066 (PMC13279077; doi:10.3389/fendo.2026.1810066)

**Supplementary Table 1. Description of missing data.**

| **Variables** | **Non-missing** | **Missing** | **Miss percentage(%)** |
| --- | --- | --- | --- |
| Age | 100494 | 0 | 0 |
| Gender | 100494 | 0 | 0 |
| Height | 100494 | 0 | 0 |
| Body weight | 100494 | 0 | 0 |
| BMI | 100494 | 0 | 0 |
| Baseline FPG | 100494 | 0 | 0 |
| ALT | 100122 | 372 | 0.370 |
| AST | 42126 | 58368 | 58.081 |
| BUN | 98186 | 2308 | 2.297 |
| Scr | 99277 | 1217 | 1.211 |
| SBP | 100481 | 13 | 0.013 |
| DBP | 100481 | 13 | 0.013 |
| HDL-C | 100494 | 0 | 0 |
| LDL-C | 100332 | 162 | 0.161 |
| TC | 100493 | 1 | 0.001 |
| TG | 100494 | 0 | 0 |
| Family histroy of Diabetes | 100494 | 0 | 0 |
| Drinking status | 27692 | 72802 | 72.444 |
| Smoking status | 27692 | 72802 | 72.444 |

**Supplementary Table 2.​ Association between SPISE index** **and the risk of incident IFG, assessed using​ Cox proportional hazards regression models in​ the original dataset**

| Variables | Crude model | | Model Ⅰ | | Model Ⅱ | |
| --- | --- | --- | --- | --- | --- | --- |
|  | HR (95%CI) | *P*-Value | HR (95%CI) | *P*-Value | HR (95%CI) | *P*-Value |
| SPISE index | 0.80 (0.80, 0.81) | < 0.001 | 0.90 (0.89, 0.91) | < 0.001 | 0.89 (0.86, 0.93) | < 0.001 |
| (SPISE index quartiles) |  |  |  |  |  |  |
| Q1 | 1.00 (Reference) |  | 1.00 (Reference) |  | 1.00 (Reference) |  |
| Q2 | 0.76 (0.73, 0.79) | < 0.001 | 0.90 (0.87, 0.94) | < 0.001 | 0.96 (0.83, 1.10) | 0.519 |
| Q3 | 0.51 (0.49, 0.54) | < 0.001 | 0.75 (0.71, 0.79) | < 0.001 | 0.85 (0.72, 1.01) | 0.059 |
| Q4 | 0.30 (0.28, 0.32) | < 0.001 | 0.58 (0.54, 0.62) | < 0.001 | 0.53 (0.42, 0.67) | < 0.001 |
| *P* for trend |  | < 0.001 |  | < 0.001 |  | < 0.001 |

Crude model: we did not adjust other covariates.

Model I: adjusted for age, gender, FPG, SBP, and DBP at baseline.

Model II: further adjusted for ALT, AST, LDL-C, TC, BUN, Scr, smoking status, drinking status, and family history of diabetes at baseline.

**Supplementary Table 3.​ Association between SPISE index and incident IFG, assessed using​ logistic​ regression models in​ the original dataset.**

| Variables | Crude model | | Model Ⅰ | | Model Ⅱ | |
| --- | --- | --- | --- | --- | --- | --- |
|  | OR (95%CI) | *P*-Value | OR (95%CI) | *P*-Value | OR (95%CI) | *P*-Value |
| SPISE index | 0.77 (0.76, 0.78) | < 0.001 | 0.87 (0.86, 0.88) | < 0.001 | 0.85 (0.82, 0.89) | < 0.001 |
| (SPISE index quartiles) |  |  |  |  |  |  |
| Q1 | 1.00 (Reference) |  | 1.00 (Reference) |  | 1.00 (Reference) |  |
| Q2 | 0.70 (0.67, 0.73) | < 0.001 | 0.81 (0.77, 0.85) | < 0.001 | 0.85 (0.73, 0.99) | 0.035 |
| Q3 | 0.44 (0.42, 0.47) | < 0.001 | 0.65 (0.61, 0.69) | < 0.001 | 0.69 (0.58, 0.83) | < 0.001 |
| Q4 | 0.25 (0.24, 0.27) | < 0.001 | 0.50 (0.47, 0.54) | < 0.001 | 0.44 (0.34, 0.56) | < 0.001 |
| *P* for trend |  | < 0.001 |  | < 0.001 |  | < 0.001 |

Crude model: we did not adjust other covariates.
Model I: adjusted for age, gender, FPG, SBP, and DBP at baseline.

Model II: further adjusted for ALT, AST, LDL-C, TC, BUN, Scr, smoking status, drinking status, and family history of diabetes at baseline.

**Supplementary Table 4.​ Association between SPISE index** **and the risk of incident IFG, assessed using​ Cox proportional hazards regression models after excluding participants with incomplete covariate data.**

| Variables | Crude model | | Model Ⅰ | | Model Ⅱ | |
| --- | --- | --- | --- | --- | --- | --- |
|  | HR (95%CI) | *P*-Value | HR (95%CI) | *P*-Value | HR (95%CI) | *P*-Value |
| SPISE index | 0.78 (0.76, 0.8) | < 0.001 | 0.87 (0.84, 0.9) | < 0.001 | 0.89 (0.86, 0.93) | < 0.001 |
| (SPISE index quartiles) |  |  |  |  |  |  |
| Q1 | 1.00 (Reference) |  | 1.00 (Reference) |  | 1.00 (Reference) |  |
| Q2 | 0.72 (0.63, 0.82) | < 0.001 | 0.83 (0.72, 0.95) | 0.009 | 0.88 (0.77, 1.02) | 0.082 |
| Q3 | 0.55 (0.48, 0.64) | < 0.001 | 0.78 (0.67, 0.91) | 0.002 | 0.86 (0.74, 1.02) | 0.076 |
| Q4 | 0.25 (0.21, 0.31) | < 0.001 | 0.49 (0.40, 0.61) | < 0.001 | 0.55 (0.44, 0.68) | < 0.001 |
| *P* for trend |  | < 0.001 |  | < 0.001 |  | < 0.001 |

Crude model: we did not adjust other covariates.
Model I: adjusted for age, gender, FPG, SBP, and DBP at baseline.

Model II: further adjusted for ALT, AST, LDL-C, TC, BUN, Scr, smoking status, drinking status, and family history of diabetes at baseline.

**Supplementary Table 5.​ Association between SPISE index** **and incident IFG, assessed using​ logistic​ regression models after excluding participants with incomplete covariate data.**

| Variables | Crude model | | Model Ⅰ | | Model Ⅱ | |
| --- | --- | --- | --- | --- | --- | --- |
|  | OR (95%CI) | *P*-Value | OR (95%CI) | *P*-Value | OR (95%CI) | *P*-Value |
| SPISE index | 0.76 (0.73, 0.78) | < 0.001 | 0.83 (0.80, 0.86) | < 0.001 | 0.85 (0.82, 0.89) | < 0.001 |
| (SPISE index quartiles) |  |  |  |  |  |  |
| Q1 | 1.00 (Reference) |  | 1.00 (Reference) |  | 1.00 (Reference) |  |
| Q2 | 0.67 (0.58, 0.78) | < 0.001 | 0.73 (0.63, 0.86) | < 0.001 | 0.79 (0.67, 0.92) | 0.003 |
| Q3 | 0.50 (0.42, 0.58) | < 0.001 | 0.65 (0.55, 0.77) | < 0.001 | 0.73 (0.61, 0.87) | < 0.001 |
| Q4 | 0.22 (0.18, 0.27) | < 0.001 | 0.38 (0.30, 0.47) | < 0.001 | 0.44 (0.35, 0.56) | < 0.001 |
| *P* for trend |  | < 0.001 |  | < 0.001 |  | < 0.001 |

Crude model: we did not adjust other covariates.
Model I: adjusted for age, gender, FPG, SBP, and DBP at baseline.

Model II: further adjusted for ALT, AST, LDL-C, TC, BUN, Scr, smoking status, drinking status, and family history of diabetes at baseline.

**Supplementary Figure 1. The linear relationship between SPISE index** **and risk of incident IFG using the original dataset.** We adjusted age, gender, FPG, SBP, DBP, ALT, AST, LDL-C, TC, BUN, Scr, smoking status, and family history of diabetes at baseline.


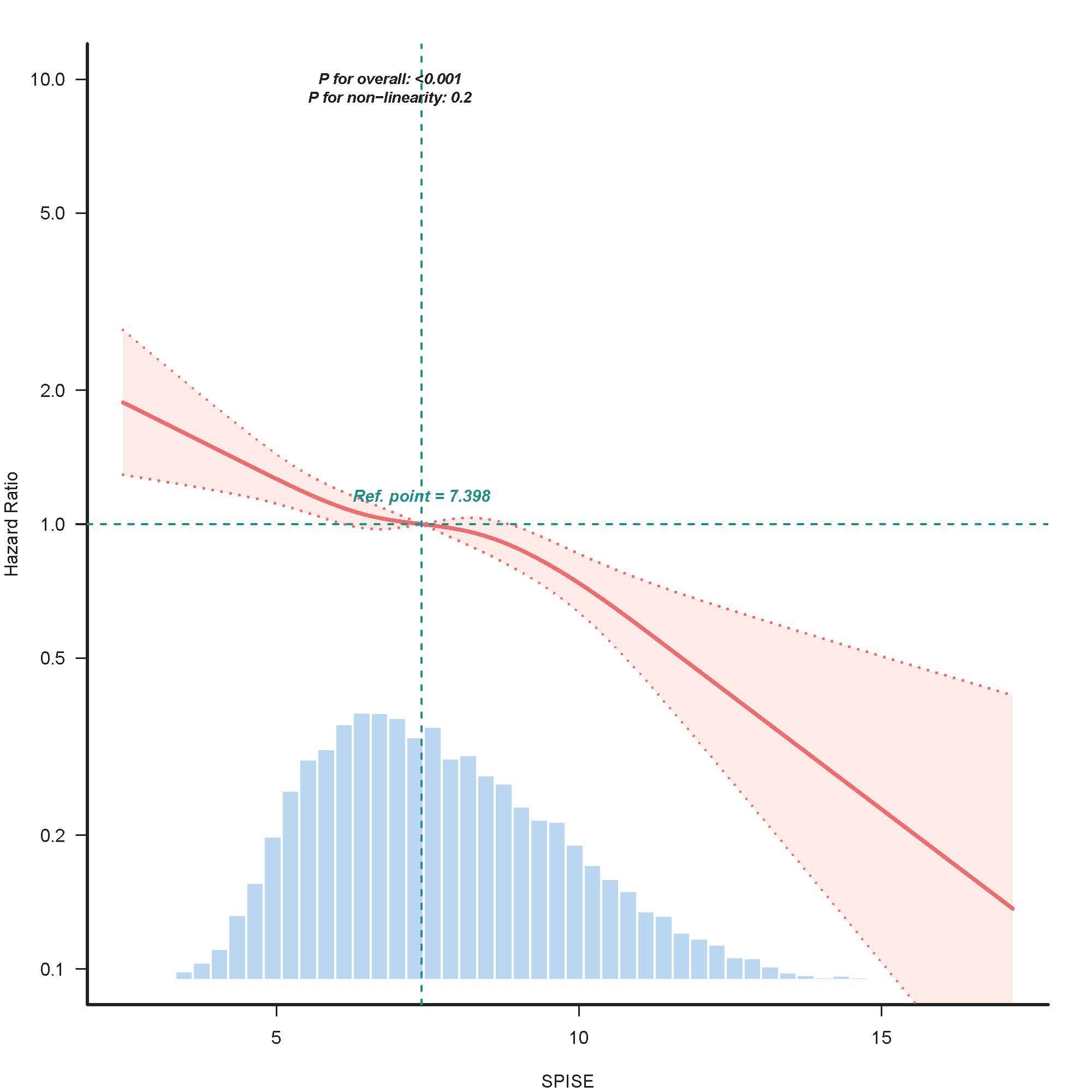


**Supplementary Figure 2. The linear relationship between SPISE index** **and risk of incident IFG using the excluding participants with incomplete covariate data.** We adjusted age, gender, FPG, SBP, DBP, ALT, AST, LDL-C, TC, BUN, Scr, smoking status, and family history of diabetes at baseline.


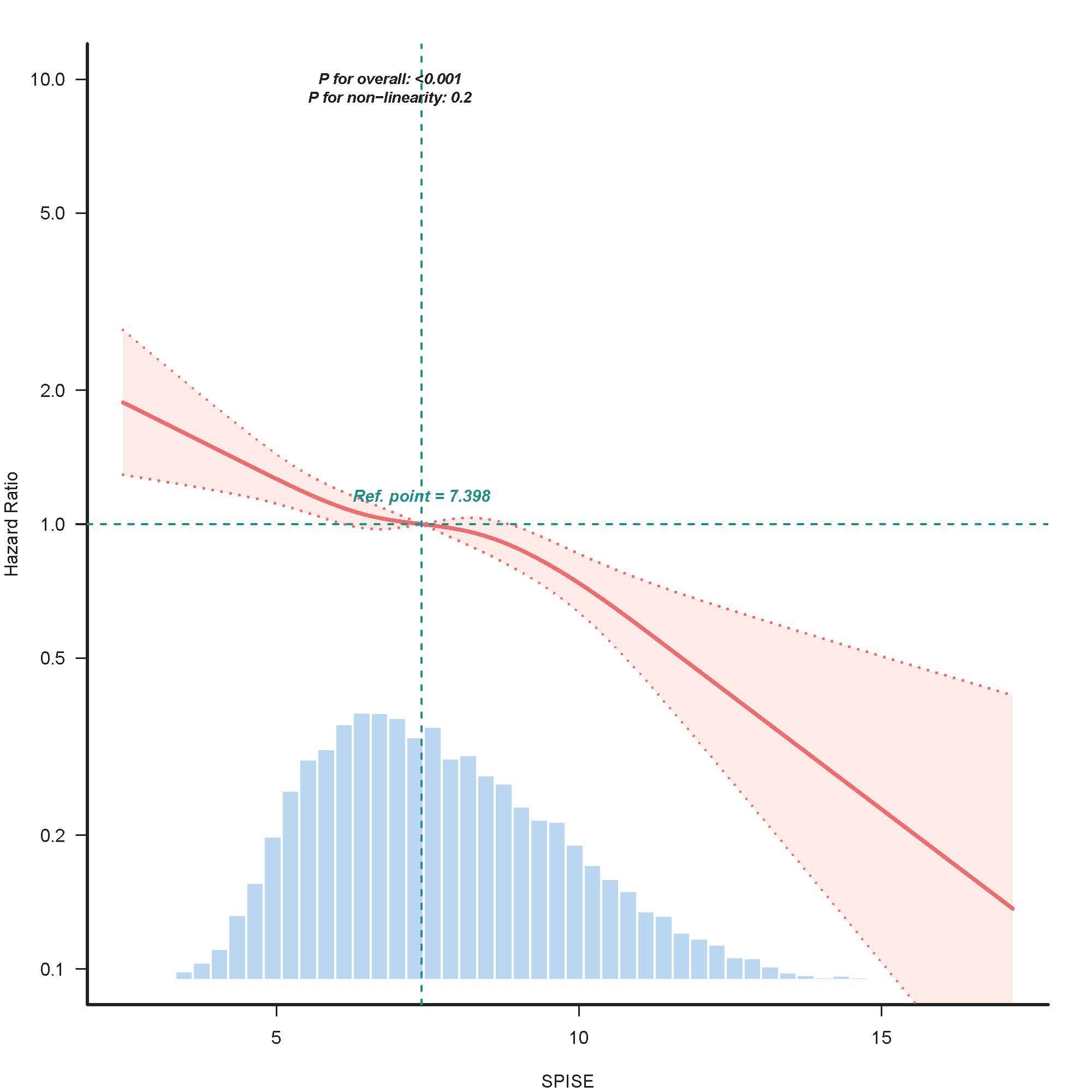

Supplement: Supplementary file 1 [file DataSheet1.docx]
